# Colon-Targeted Trans-Cinnamic Acid Ameliorates Rat Colitis by Activating GPR109A

**DOI:** 10.3390/pharmaceutics15010041

**Published:** 2022-12-22

**Authors:** Changyu Kang, Jaejeong Kim, Sanghyun Ju, Heeyeong Cho, Hyun Young Kim, In-Soo Yoon, Jin-Wook Yoo, Yunjin Jung

**Affiliations:** 1 College of Pharmacy, Pusan National University, Busan 46241, Republic of Korea; 2 Biotechnology & Therapeutic Division, Korea Research Institute of Chemical Technology, Daejeon 34114, Republic of Korea; 3 Department of Medicinal and Pharmaceutical Chemistry, Korea University of Science and Technology, 141 Gajeong-ro, Yuseong, Daejeon 34114, Republic of Korea

**Keywords:** cinnamic acid, colon-specific drug delivery, inflammatory bowel disease, GPR109A, prodrug

## Abstract

We designed colon-targeted trans-cinnamic acid (tCA) and synthesized its conjugates with glutamic acid (tCA-GA) and aspartic acid (tCA-AA). We evaluated the anti-colitic activity of colon-targeted tCA using a dinitrobenzenesulfonic acid-induced rat colitis model. The conjugates lowered the distribution coefficient and Caco-2 cell permeability of tCA and converted to tCA in the cecum, with higher rates and percentages with tCA-GA than with tCA-AA. Following oral gavage, tCA-GA delivered a higher amount of tCA to the cecum and exhibited better anti-colitic effects than tCA and sulfasalazine (SSZ), which is the current treatment for inflammatory bowel disease. In the cellular assay, tCA acted as a full agonist of GPR109A (EC50: 530 µM). The anti-colitic effects of tCA-GA were significantly compromised by the co-administration of the GPR109A antagonist, mepenzolate. Collectively, colon-targeted tCA potentiated the anti-colitic activity of tCA by effectively activating GPR109A in the inflamed colon, enabling tCA to elicit therapeutic superiority over SSZ.

## 1. Introduction

Inflammatory bowel disease (IBD), comprising ulcerative colitis (UC) and Crohn’s disease, is a chronic and recurrent inflammation of the gastrointestinal tract [1]. Currently, no curative medicine is available for IBD. The medical aim of anti-IBD drugs is to induce and maintain remission for a long time [2,3]. Anti-IBD drugs, including aminosalicylates, glucocorticoids, and immunosuppressants, have disadvantages such as low efficacy (aminosalicylates) and serious side effects following long-term therapy [4,5]. Recently, biologics, including anti-tumor necrosis factor, anti-integrin, and anti-interleukin (IL)-12 and IL-23 agents, introduced into anti-IBD pharmacotherapy, have proven to be effective in patients with IBD resistant to conventional drugs [6,7]. However, biopharmaceuticals are not free of side effects, and therapeutic tolerance and resistance can be established due to the formation of antibodies against the biologics. High medication costs, poor patient compliance due to parenteral administration, and tolerance development are also major drawbacks to the lifelong use of biologics [8]. Therefore, an unmet medical need exists for the development of small-molecule anti-IBD drugs that improve the therapeutic and toxicological properties of conventional drugs.

Natural products are major resources for the development of new drugs [9]. During our search for a candidate natural product to develop an anti-IBD drug, cinnamic acid (CA) was identified as a lead to meet our conditions, including “abundant”, “safe”, “anti-inflammatory”, and “structure modifiable to a colon-targeted prodrug”. CA (IUPAC name: 3-phenylprop-2-enoic acid) is a natural compound found abundantly in plants, such as cinnamon (*Cinnamomum cassia* and *Cinnamomum zeylanicum*) and *Panax ginseng*, as well as in human dietary products, such as fruits, whole grains, vegetables, and honey [10]. The acrylic acid group substituted on the aromatic ring, so CA exists in either a trans or cis-form, with the trans-form being more common than the cis-form. CA is generally safe and FDA-approved as a food additive and adjuvant [10,11]. CA reportedly exhibits various biological activities, including anti-inflammatory activity [11,12]. Further, CA and its derivatives are beneficial for periodontitis and intestinal inflammation [13,14]. Trans-CA (tCA), produced in the large intestine via microbial metabolism of phenylalanine and polyphenolic compounds in food, enhances intestinal barrier function and modulates gut microbiota, thereby favoring health. tCA also serves as a major intermediate compound in the microbial synthesis of antibiotics, anti-fungal, or anti-viral compounds, such as 5-dihydroxy-4-isopropyl-stilbene, enterocin, and soraphen [15,16].

Colon-specific drug delivery (CSDD) is employed to enhance the therapeutic activities and safety of drugs, especially for the treatment of colonic diseases, such as IBD [17,18]. In general, the benefit of this delivery technique includes increased drug availability in the large intestine while decreasing systemic absorption of the drug; the drug is ultimately delivered to the target site, without significant loss in the upper intestine, due to systemic absorption and pre-systemic metabolism [17,19]. The prodrug approach is one of the major strategies for accomplishing the pharmaceutical goal, CSDD [19]. Colon-specific prodrugs using small molecular carriers are widely adopted for CSDD, and those currently used to treat IBD include sulfasalazine (SSZ), olsalazine, and balsalazide, which are mesalazine (5-aminosalicylic acid, 5-ASA) azo-linked to various small molecular carriers [18,20].

tCA possesses a carboxylic group linked to a colon-targeted carrier, such as amino acids via an amide, which can be microbially cleaved to release tCA in the large intestine while remaining intact in the upper intestine [21]. In this study, tCA-glutamic acid (tCA-GA) and tCA-aspartic acid (tCA-AA) were synthesized as colon-targeted prodrugs of tCA, and their colon specificity was tested. The anti-colitic activity of tCA-GA, whose conversion to tCA via microbes occurs faster than that of tCA-AA, was evaluated in a dinitrobenzene sulfonic acid (DNBS)-induced rat colitis model and compared with that of SSZ. In addition, based on the structural similarity of tCA to the GPR109A agonist, butyric acid [22]. A potential molecular mechanism for tCA-mediated anti-colitic activity was investigated by in vitro and in vivo experiments.

## 2. Materials and Methods

### 2.1. Materials

*L*-Glutamic acid dimethyl ester hydrochloride, tCA, 1,1′-carbonyldiimidazole (CDI), DNBS, nicotinic acid (NA), trimethylamine (TEA), and SSZ were purchased from Tokyo Chemical Industry (Tokyo, Japan). *L*-Aspartic acid dimethyl ester hydrochloride, lipopolysaccharides (LPS from *Escherichia coli* 0111:B4), and mepenzolate (MPZ) were purchased from Sigma Chemical Co., Inc. (St. Louis, MO, USA). The solvents used for synthesis such as ethyl acetate (EA) and acetonitrile (ACN) were purchased from Junsei Chemical Co. (Tokyo, Japan) and DAEJUNG Chemicals & Metals Co., Ltd. (Gyeonggi-do, Republic of Korea). Cytokine-induced neutrophil chemoattractant-3 (CINC-3) and interleukine-10 (IL-10) were quantified using enzyme-linked immunosorbent assay (ELISA) kits obtained from R&D Systems (Minneapolis, MN, USA). The chemicals and solvents were used without additional purification. For the identification of structures, conjugates synthesized were subjected to infrared (IR), proton nuclear magnetic resonance (^1^H-NMR), and mass analysis. A Varian FT-IR spectrophotometer (Varian, Palo Alto, CA, USA), a Varian AS 500 NMR spectrophotometer (Varian), and a mass spectrometer (LCMS-8050, Shimadzu Co., Kyoto, Japan) were used for the structural analysis. Tetramethylsilane was used as an internal reference compound to present the chemical shifts in the NMR spectra.

### 2.2. Cell Culture

Human colon carcinoma HCT116 and Caco-2 cells and murine macrophages RAW 264.7 cells were grown in Dulbecco’s modified Eagle’s medium (DMEM, HyClone, Logan, UT, USA), supplemented with fetal bovine serum (100 mL/L, FBS, HyClone) and penicillin/streptomycin (10 mL/L, HyClone). Gα16-coupled CHO cells (CHO/Gα16) were maintained in Ham’s F-12 culture medium (HyClone), supplemented with FBS (100 mL/L) and penicillin/streptomycin (10 mL/L).

### 2.3. Synthesis of N-Cinnamoyl-L-Glutamic Acid (tCA-GA) and N-Cinnamoyl-L-Aspartic Acid (tCA-AA)

The carboxylic group in tCA (148 mg, 1 mmol) was activated by reaction with CDI (201 mg, 1.3 mmol) dissolved in 10 mL ACN for 30 min, followed by the addition of *L*-glutamic acid dimethyl ester hydrochloride (420 mg, 2 mmol) and TEA (2 mL). The reaction mixture was stirred at 20–25 °C for 24 h and, then, was evaporated to remove the reaction solvent. The residue dissolved in EA was washed three times with 0.1 M HCl and 5% Na_2_CO_3_ solution. Following evaporation, 8 mL of 0.5 M NaOH solution was added to the residue and stirred at 40 °C for 3 min, and then, it was acidified with a 1.0 M HCl solution and extracted using EA. The organic layer was dried over anhydrous Na_2_SO_4_ and evaporated to obtain tCA-GA as a white powder. tCA-AA was synthesized using *L*-aspartic acid dimethyl ester hydrochloride; the synthetic method was the same as that used for tCA-GA. tCA-GA (M.W.: 277.28); Yield: 72%; mp: 203 °C; IR (nujol mull), ν_max_ (cm^−1^): 1698, 1667 (C=O, -COOH), 1622 (C=O, -CONH); ^1^H-NMR (DMSO-*d*6): δ = 8.40 (d, *J* = 7.9 Hz, 1H), 7.67–7.50 (m, 2H), 7.50–7.13 (m, 4H), 6.73 (d, *J* = 15.8 Hz, 1H), 4.36 (td, *J* = 8.9, 5.1 Hz, 1H), 2.51 (dd, *J* = 3.6, 1.8 Hz, 1H), 2.09–1.99 (m, 1H), 1.84 (dd, *J* = 14.7, 8.8, 6.2 Hz, 1H). [M-H]^-^: *m/z* = 276.15. tCA-AA (M.W.: 263.25); Yield: 68%; mp: 164 °C; IR (nujol mull), ν_max_ (cm^−1^): 1703, 1660 (C=O, -COOH), 1628 (C=O, -CONH); ^1^H-NMR (DMSO-*d*6): δ = 8.44 (d, *J* = 8.0 Hz, 1H), 7.58 (d, *J* = 7.1 Hz, 2H), 7.48-7.25 (m, 4H), 6.76 (d, *J* = 15.8 Hz, 1H), 4.68 (dd, *J* = 7.3, 5.6 Hz, 1H), 2.80–2.71 (m, 1H), 2.68 (dd, *J* = 16.6, 7.2 Hz, 1H). [M-H]^−^: *m/z* = 262.15.

### 2.4. High-Performance Liquid Chromatography Analysis

For high-performance liquid chromatography (HPLC) analysis, a Gilson HPLC system (Gilson, Middleton, WI, USA) was used. Samples (20 μL) filtrated through membrane filters (0.45 μm, Revodix, Gyeonggi-do, South Korea) were applied to a symmetric C_18_ column (Hector, Theale, Berkshire, UK; 250 × 4.6 mm, 5 μm) using a model 234 auto-injector (Gilson). tCA, tCA-GA, and tCA-AA were separated at a flow rate of 1.0 mL/min in a mobile phase comprising methanol, acetonitrile, and 1% acetic acid solution (1.5:2.2:6.3), which was monitored at 277 nm using a 151 variable UV detector (Gilson). The retention times of tCA, tCA-GA, and tCA-AA were 8.1, 17.3, and 18.5 min, respectively.

### 2.5. Distribution Coefficient and Chemical Stability

Distribution coefficients (log *D*_6.8_) of tCA, tCA-AA, and tCA-GA were measured as described previously [23]. The concentrations of drugs were determined using a UV-Vis spectrophotometer (Shimadzu, Tokyo, Japan) at 277 nm, and log *D*_6.8_ was calculated using the equation reported previously [23]. The chemical stabilities of tCA-GA and tCA-AA were analyzed in HCl-NaCl buffer (pH 1.2) and pH 6.8 isotonic phosphate buffer, representing the physiological pH of the stomach and small intestine, respectively. Each drug (0.1 mM) dissolved in the buffers was incubated for at least 12 h, and change in the concentrations of the drugs was monitored using HPLC analysis.

### 2.6. Cell Permeability Assay

Caco-2 cells were seeded at 1 × 10^5^ cells per insert in Transwell inserts with a pore size of 0.4 μm (SPL Inc., Houston, TX, USA). DMEM, containing 10% FBS and 1% penicillin/streptomycin, was added to the basolateral (well) (1 mL) and apical (insert) compartments (0.2 mL). The cells were grown until the trans-epithelial electrical resistance value (EMD Millipore, Billerica, MA, USA) of the cell monolayer reached 2000 Ω cm^2^. After removing the culture medium, tCA and its derivatives (1.2 mM) dissolved in DMEM (0.2 mL) without phenol red and were placed on the apical side of the cell monolayer. At appropriate time intervals, the concentrations of the drugs on the basolateral side filled with DMEM (1.0 mL) were determined using HPLC.

### 2.7. Conversion of the Amino Acid Conjugates to tCA in the Contents of the GI Tract

Male Sprague–Dawley (SpD) rats (250–260 g) were killed using CO_2_ gas. The proximal small intestinal, distal small intestinal, and cecal contents were collected separately and suspended in pH 6.8 isotonic phosphate buffer to prepare a 20% *w*/*v* suspension. tCA-GA and tCA-AA (10 mM) dissolved in pH 6.8 isotonic phosphate buffer (4 mL) were added to the suspension (4 mL) and incubated at 37 °C. To maintain anaerobic conditions, the collection of cecal contents and incubation of the conjugates in the cecal contents were conducted in a nitrogen gas bag (Sigma-Aldrich, Burlington, MA, USA). At appropriate time intervals, a portion of the mixture (0.5 mL), pipetted using a wide-orifice tip, was centrifuged in a microtube. After acidification (with 1.0 M HCl) of the supernatant (0.3 mL), EA was added and vigorously mixed. The organic layer (0.2 mL) was retrieved and evaporated. The residue, dissolved in the mobile phase (0.2 mL), was filtered and subjected to HPLC analysis. Centrifugation was conducted at 10,000× *g* for 10 min at 4 °C.

### 2.8. Calcium Mobilization Assay

GPR109A-dependent calcium mobilization was analyzed using a Fluo-4 NW calcium assay kit (Molecular Devices, Sunnyvale, CA, USA) in CHO/Gα16 cells stably expressing GPR109A. Calcium mobilization assay was performed as described previously [24]. All experiments were performed in duplicate and repeated more than twice. The EC_50_ values were obtained from nonlinear regression analysis using GraphPad Prism version 5.0 for Windows (GraphPad Software, San Diego, CA, USA).

### 2.9. Animals

The seven-week-old male SpD rats (Samtako Bio Korea, Kyeong-gi-do, Republic of Korea) were acclimated (for at least three days) in the PNU Laboratory Animal Center at Pusan National University, Busan, Republic of Korea, where temperature, humidity, and dark (12 h)/light (12 h) cycle were controlled. The Pusan National University Institutional Animal Care and Use Committee (PNU–IACUC) approved the animal protocol, satisfying the IACUC protocol requirement (Approval No: PNU-2021-2942, Approval Date: 23 March 2021).

### 2.10. Animal Experimental Design

For an animal experiment to evaluate the anti-colitic effects of drugs, six groups (n = 5 rats per group) were made and treated via oral gavage as follows: normal group, 1.0 mL of phosphate buffered saline (pH 7.4, PBS); colitis group, 1.0 mL of PBS; tCA-treated colitis group, tCA (30 mg/kg) in 1.0 mL of PBS; tCA-GA-treated colitis group (L), tCA-GA (28 mg/kg, equivalent to 15 mg/kg of tCA) in 1.0 mL of PBS; tCA-GA-treated colitis group (H), tCA-GA (56 mg/kg, equivalent to 30 mg/kg of tCA) in 1.0 mL of PBS; SSZ-treated colitis group, SSZ (30 mg/kg) in 1.0 mL of PBS. For the other experiment to evaluate GPR109A-dependent anti-colitic effects of tCA-GA, five groups (n = 5 rats per group) were made and treated via oral gavage as follows: normal group, 1.0 mL of PBS; colitis group, 1.0 mL of PBS; tCA-GA-treated colitis group, tCA-GA (28 mg/kg) in 1.0 mL of PBS; tCA-GA + MPZ-treated colitis group, tCA-GA (28 mg/kg) and MPZ (2 mg/kg) in 1.0 mL of PBS; MPZ-treated colitis group, MPZ (2 mg/kg) in 1.0 mL of PBS.

### 2.11. Analysis of Drug Concentration in the Cecum

Male SpD rats were not fed except for water for 24 h. tCA (30 mg/kg) or tCA-GA (56 mg/kg, equivalent to 30 mg/kg of tCA) in PBS (1.0 mL) was administered to rats. Cecal contents were obtained at 2, 4, and 6 h after oral gavage, and pH 6.8 isotonic phosphate buffer was added to prepare a 10% suspension of cecal contents. The suspension was subjected to centrifugation at 10,000× *g* for 10 min at 4 °C, and the supernatant (0.2 mL) in a new microtube was acidified with 1.0 M HCl, followed by extraction with EA (0.3 mL). The organic layer (0.2 mL) was retrieved into a new microtube and subjected to complete evaporation. The residue dissolved in the mobile phase (0.2 mL) was filtered through a membrane filter (0.45 μm). The filtrates (20 μL) were subjected to HPLC analysis to determine concentrations of each drug in samples. 

### 2.12. DNBS-Induced Rat Colitis and Evaluation of the Anti-Colitic Effects

DNBS-mediated colitis was induced in rats as described previously [23,25]. Additionally, 3 days after the induction of colitis, the drugs were administered once per day for 6 days as in Section 2.10. The rats were killed to obtain the inflamed distal colon. The colonic damage score (CDS), assessing pathological state in colon tissue and neighbor organs ascribed to DNBS-induced inflammation, was calculated according to the criteria set previously [26,27]. For histological evaluation, fixed colon tissues were embedded in paraffin, sections with a thickness of 4 μm were cut and stained with hematoxylin and eosin (H&E). The sections were examined under a light microscope (Olympus BX43, Olympus Co., Tokyo, Japan). Myeloperoxidase (MPO) activity was measured as described previously [23,27].

### 2.13. Western Blot Analysis

Changes in levels of the proteins were monitored using Western blotting. Cell lysates and tissue lysates of the distal colon were prepared for Western blot analysis as described previously [18,28]. To determine the protein concentrations in the lysates, bicinchoninic acid reagent (Thermo Fisher Scientific, Waltham, MA, USA) was used according to the manufacturer’s instructions. SDS-PAGE (10 or 7.5% gels) was used to separate and analyze the proteins in the sample lysates. Anti-cyclooxygenase (COX-2, sc-365374, Santa Cruz Biotechnology, Dallas, TX, USA) and anti-inducible nitric oxide synthase (iNOS) antibodies (sc-7271, Santa Cruz Biotechnology) were used to detect COX-2 and iNOS proteins, respectively. Horseradish peroxidase-conjugated secondary antibodies (Santa Cruz Biotechnology) were used to label the primary antibodies. The protein bands in membranes were visualized using the SuperSignal chemiluminescence substrate (Thermo Fisher Scientific, Waltham, MA, USA). As a loading control, α-tubulin was detected using an anti-α-tubulin antibody (Santa Cruz Biotechnology). Intensity of Western blots was quantified using Image Lab software (version 5.2 build 14, Bio-Rad, Hercules, CA, USA). The mean values (n = 3 for cell experiments, n = 5 for animal experiments) were shown under the blots in figures. Original images of Western blots are shown in Supplementary Data S1.

### 2.14. ELISA for CINC-3 and IL-10

The distal colon samples (0.1 g) obtained from rats were minced in a potassium phosphate buffer (pH 6.0, 1.0 mL), homogenized using a T10 basic/IKA homogenizer (ULTRA-TURRAX^®^, Staufen im Breisgau, Germany), and centrifuged at 10,000× *g* for 10 min at 4 °C. The levels of CINC-3 and IL-10 were determined in the samples using ELISA kits, according to the manufacturer’s instructions.

### 2.15. Data Analysis

Statistical analysis of data was performed using one-way analysis of variance (ANOVA), followed by Tukey’s honestly significant difference (HSD) test or Mann–Whitney *U* test (for CDS). Difference at *p* < 0.05 is considered statistically significant.

## 3. Results

### 3.1. Synthesis of Trans-Cinnamic Acid Coupled with Acidic Amino Acids via an Amide Bond

**Figure 1:**
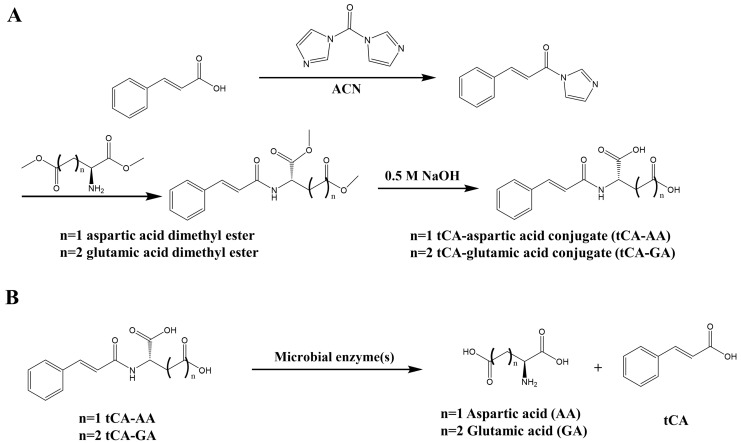
Synthesis of tCA-GA and tCA-AA and the colonic activation of the conjugates. (**A**) Synthetic scheme of tCA-GA and tCA-AA; (**B**) proposed scheme for the colonic activation of the conjugates.

tCA was coupled with the acidic amino acids, glutamic acid, and aspartic acid, via an amide bond, to yield tCA-GA and tCA-AA, respectively, as shown in Figure 1A. The proposed scheme for colonic activation of tCA-GA and tCA-AA is shown in Figure 1B. To verify the synthesis of the final products, the IR and ^1^H-NMR spectra were recorded for tCA-GA and tCA-AA. As shown in Supplementary Data S2A, in the IR spectra, the carbonyl bands for the two carboxylic groups of the acidic amino acids were observed at 1698 and 1667 cm^−^^1^ (for tCA-GA), as well as 1703 and 1660 cm^−^^1^ (for tCA-AA), while the carbonyl bands ascribed to the amide groups formed by the conjugation of tCA and the amino acids were observed at 1622 (for tCA-GA) and 1628 cm^−^^1^ (for tCA-AA). In the ^1^H-NMR spectra, the signals of the protons derived from tCA, and the amino acids were detected (Supplementary Data S2B). The mass spectra of tCA-GA and tCA-AA displayed molecular peaks that corresponded to each conjugate (Supplementary Data S2C).

### 3.2. tCA-GA Is Delivered and Converted to tCA in the Large Intestine of Rats

**Figure 2:**
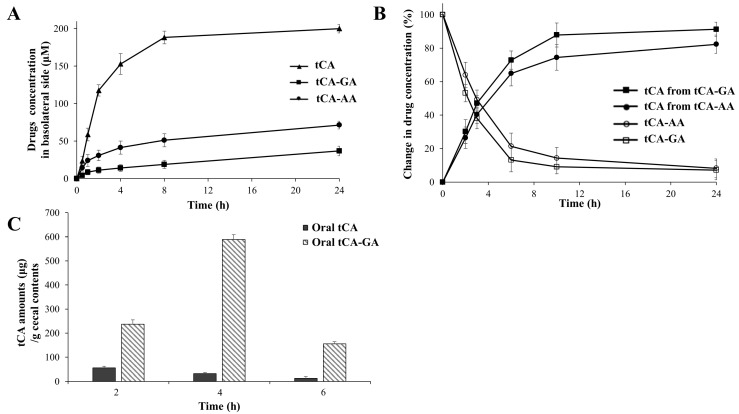
tCA, conjugated with acidic amino acids, acts as a colon-targeted prodrug of tCA. (**A**) tCA-GA, tCA-AA, and tCA (1.2 mM, 200 µL) dissolved in medium were added to the apical side of the Caco-2 cell monolayer and incubated for 24 h. At appropriate time intervals, the concentrations of each drug were determined in the basolateral side filled with medium (1 mL) using HPLC. (**B**) tCA-GA (1 mM) was incubated with the cecal contents suspended in PBS (pH 6.8, 10%) under nitrogen. The concentrations of tCA-GA and tCA were analyzed using HPLC at appropriate time intervals. The same experiment was performed with tCA-AA (1 mM). (**C**) tCA (30 mg/kg) or tCA-GA (56.1 mg/kg, equivalent to 30 mg/kg of tCA) suspended in PBS (pH 7.4, 1 mL) was administered orally to rats. The rats were killed 2, 4, and 6 h later, and the concentrations of tCA in the cecum were analyzed using HPLC. The data in A, B, and C are presented as mean ± SD (n = 5).

The colon specificities of tCA-GA and tCA-AA were assessed in vitro and in vivo. The amino acid conjugation to tCA lowered the distribution coefficient (DC) of tCA (2.23) to −0.98 (tCA-GA) and −0.61 (tCA-AA) in an n-octanol/buffer (pH 6.8) system, indicating that the amino acid conjugation increased the hydrophilicity of tCA. To determine whether lowering the DC deterred passive transport via the intestinal epithelial layer, the amino acid conjugates were subjected to a cell permeability test using a Caco-2 cell monolayer and compared with tCA. As shown in Figure 2A, the amino acid conjugation of tCA substantially delayed the permeation of tCA through the cell monolayer, with a greater delay obtained with tCA-GA than with tCA-AA. To determine the stability of the amino acid conjugates in the stomach and small intestine, tCA-GA and tCA-AA were incubated in buffers (pH 1.2, 6.8), and the small intestinal contents were collected from rats. In the incubated media, no notable changes in the concentrations of the amino acid conjugates were observed for up to 10 h. In contrast, when tCA-GA and tCA-AA were incubated in the cecal contents under nitrogen, the concentrations of tCA increased as the amino acid conjugates disappeared, as shown in Figure 2B. The release percentages of tCA from tCA-GA were approximately 30% at 2 h and 87% at 10 h, whereas those of tCA from tCA-AA were approximately 26% at 2 h and 74% at 10 h. In parallel, the conversion rate of tCA-GA to tCA was higher than that of tCA-AA to tCA. To confirm whether the conversion of the amino acid conjugates occurred via microbial enzymes, a cleavage test was conducted with autoclaved cecal contents, as reported previously [29]. The amino acid conjugates remained intact for up to 24 h. Finally, to determine whether the conjugates could effectively deliver tCA to the large intestine, tCA-GA, which had a greater conversion rate, was administered to rats via oral gavage (oral tCA-GA), and tCA was detected in the cecum at appropriate time intervals. To enable comparison, the same experiment was conducted using tCA. As shown in Figure 2C, oral tCA-GA accumulated greater amounts of tCA than oral tCA at all time points. The maximal cecal concentration was approximately 588 and 55 µg/g cecal content with oral tCA-GA and tCA, respectively.

### 3.3. tCA-GA Potentiates the Anti-Colitic Activity of tCA and Is More Effective Than SSZ

**Figure 3:**
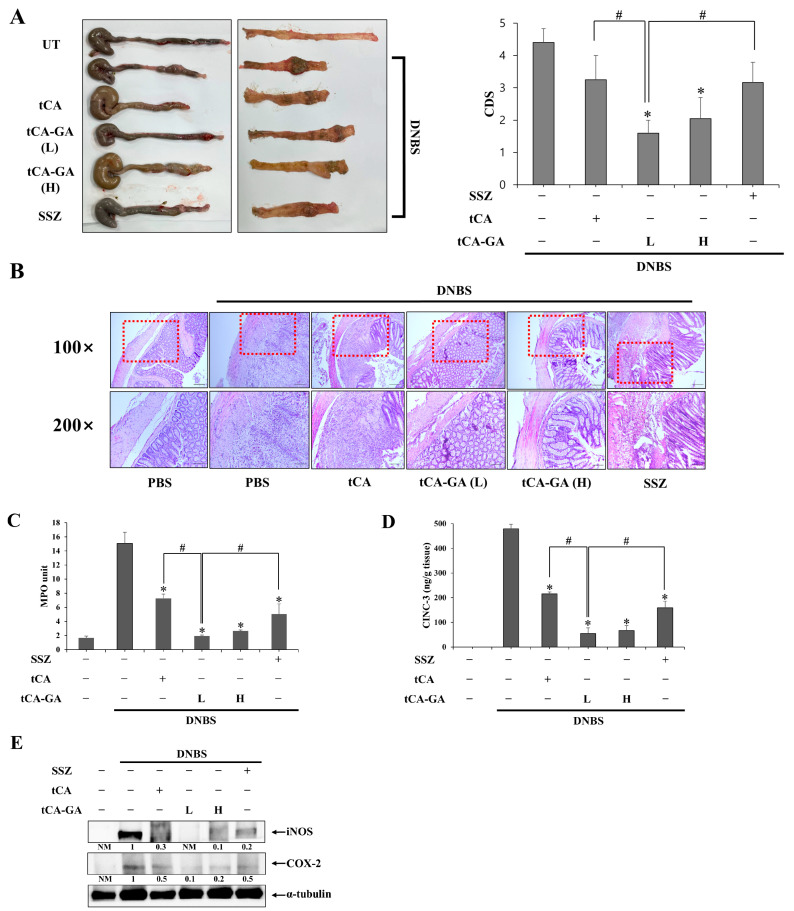
tCA-GA potentiates the anti-colitic activity of tCA and is more effective than SSZ at ameliorating rat colitis. DNBS-mediated colitis was induced for 3 days, and tCA (30 mg/kg), SSZ (30 mg/kg) or tCA-GA [equivalent to 15 mg/kg (L), as well as 30 mg/kg (H) of tCA] suspended in PBS (1.0 mL) was administered orally to colitic rats once per day. One day after the last treatment, the rats were killed to evaluate anti-colitic effects of the drugs. (**A**) Left panel: Photos of the distal colons of rats where serosal and luminal sides are shown separately. Right panel: Overall colonic damage was scored for each group and presented as colonic damage score (CDS). * α < 0.05, vs. DNBS control (**B**) H&E staining was performed with the colon sections of rats subjected to various treatments. Upper panel: Representative images of 100× magnification (scale bar: 200 µm). Lower panel: Representative images of 200× magnification for the dotted boxes in the upper panel (scale bar: 100 µm). In the inflamed distal colons (4.0 cm), (**C**) myeloperoxidase (MPO) activity was measured in addition to determining the levels of (**D**) CINC-3, (**E**) iNOS, and COX-2 using an Elisa kit and Western blotting. A loading control (α-tubulin) was used for Western blot analysis of COX-2 and iNOS. The data in (**A**,**C**,**D**) are presented as mean ± SD (n = 5). Statistical significance was analyzed using a one-way ANOVA followed by Tukey’s HSD test, * *p* < 0.05, vs. DNBS control. ^#^ *p* < 0.05.

We determined whether tCA elicited a beneficial effect on intestinal inflammation and whether the colon-targeted delivery of tCA improved the therapeutic activity of tCA. Accordingly, tCA (30 mg/kg) or tCA-GA half-equivalent or equivalent to the tCA dose was administered orally to rats with DNBS-induced colitis. To enable a comparison with the current anti-IBD drug, SSZ (30 mg/kg), was also administered orally. The colitic rats were medicated once per day for 6 days, and the anti-colitic effects of the drugs were assessed. As shown in Figure 3A (left panel showing the photos of distal colons), the colonic instillation of DNBS induced severe damage in the colon of rats, showing pathological lesions, such as hemorrhagic ulcers, edemas, strictures, adhesions with neighboring organs, and shortenings of the colon length. Change in the colon length is presented as a bar graph in Supplementary Data S3A. Treatment with tCA or tCA-GA ameliorated colonic damage. Further, both doses of tCA-GA were significantly more effective than tCA at mitigating pathological changes. A low dose (L) of tCA-GA was slightly more effective than a high dose (H). tCA-GA was also more effective than SSZ, whereas tCA was as effective as SSZ. In Figure 3A (right panel), colonic damage is scored according to the criteria shown in Supplementary Data S4. H&E staining of the inflamed distal colons showed that both doses of tCA-GA recovered the mucosal layer of the distal colon, which was greater than that of tCA and SSZ (Figure 3B). Colonic inflammation was assessed by measuring MPO activity and the levels of inflammatory mediators (CINC-3, COX-2, and iNOS) in the inflamed distal colon of rats. As shown in Figure 3C–E, MPO activity and inflammatory mediator levels were elevated by DNBS-induced colonic inflammation. Consistent with the results shown in Figure 3A, tCA and tCA-GA lowered MPO activity (Figure 3C), as well as the levels of CINC-3 (Figure 3D) and iNOS and COX-2 proteins (Figure 3E). Further, tCA-GA was confirmed to be more effective than tCA and SSZ, whereas tCA was as effective as SSZ. No significant difference in anti-inflammatory effects was found between the administration of the high and low doses of tCA-GA.

### 3.4. tCA Is an Agonist of GPR109A

**Figure 4:**
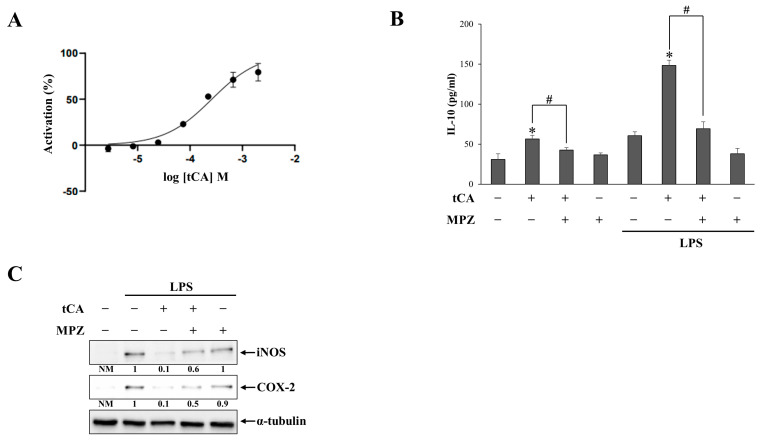
tCA is a GPR109A agonist. (**A**) Agonistic activity of tCA on GPR109A was measured using a calcium mobilization assay. Nicotinic acid (10 μM) was used as a reference agent whose activity of GPR109A is set as 100%, and tCA activity is presented as the percentage activation. (**B**) RAW264.7 cells were 1 h-pretreated with MPZ (100 μM) and tCA (5 mM) and, then, were challenged with lipopolysaccharide (LPS) for 24 h. The cell supernatants were subjected to ELISA, using a kit to determine the levels of IL-10. (**C**) RAW264.7 cells, pretreated with MPZ (100 μM) and tCA (5 mM) for 1 h, were challenged with LPS for 24 h. The levels of iNOS and COX-2 proteins were analyzed using Western blotting. α-Tubulin was used as a loading control. NM: not measurable. The data in B are presented as mean ± SD (n = 5). Statistical significance was analyzed using a one-way ANOVA, followed by Tukey’s HSD test, * *p* < 0.05, vs. Control. ^#^ *p* < 0.05.

To investigate the molecular mechanism of the tCA-GA-mediated anti-colitic activity, we determined whether tCA acts as an agonist of GPR109A. This hypothesis was based on the structural similarity of tCA to the GPR109A agonist, butyric acid (Supplementary Data S5), and the therapeutic benefits of GPR109A agonists in rat colitis [28,30,31]. tCA was subjected to a cell-based calcium mobilization assay using CHO/Gα16 cells stably transfected with the GPR109A cDNA. NA, a full agonist of GPR109A, was used as the reference agonist. As shown in Figure 4A, tCA increased calcium mobilization in a concentration-dependent manner, and the activity of 2 mM tCA was equivalent to that of 10 µM NA. This result indicated that tCA is an agonist of GPR109A, although its potency (EC_50_: 530 µM) was markedly lower than that of NA (EC_50_: 52 nM). To verify the tCA activation of GPR109A, the murine macrophage RAW 264.7 cells, pre-stimulated with LPS, were treated with 2 mM tCA in the presence or absence of the GPR109A antagonist, MPZ, and levels of the anti-inflammatory cytokine, IL-10, in the cell culture supernatants were determined. A typical anti-inflammatory response mediated by GPR109A activation is the induction of IL-10 [28]. As shown in Figure 4B, tCA increased the level of IL-10 and further increased LPS-induced elevated IL-10 levels, which were significantly suppressed by MPZ. As GPR109 activation inhibits NFκB activity [28], we also determined whether tCA prevented LPS-mediated induction of COX-2 and iNOS proteins, products of NFκB target genes, in RAW 264.7 cells and whether these effects were compromised by MPZ. As shown in Figure 4C, tCA reduced the levels of COX-2 and iNOS, and MPZ undermined the suppressive effects of tCA. 

### 3.5. Anti-Colitic Effects of tCA-GA Are Blunted by GPR109A Antagonist

**Figure 5:**
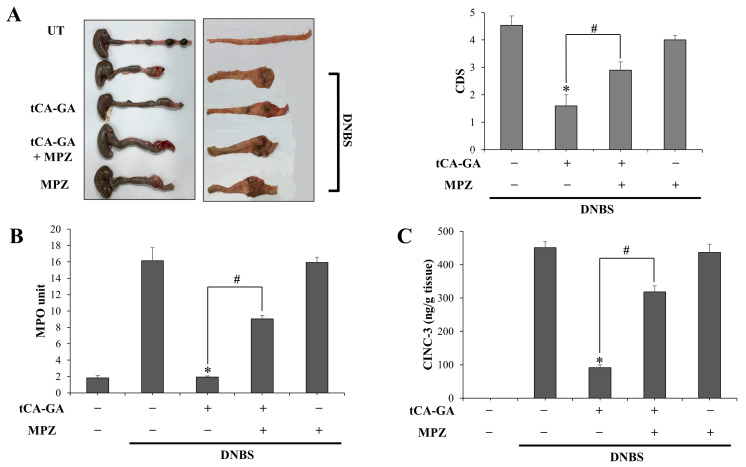

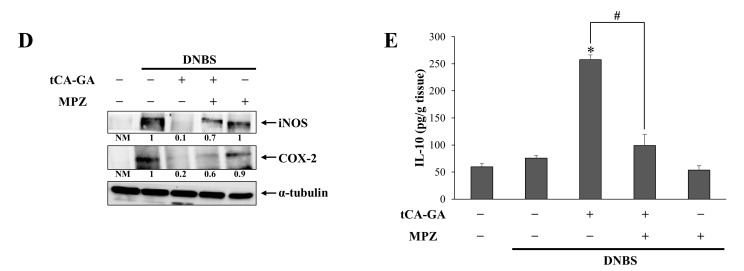
Anti-colitic effects of tCA-GA are blunted by a GPR109A antagonist. DNBS-mediated colitis was induced for 3 days, and tCA-GA (28 mg/kg), tCA-GA (28 mg/kg) + MPZ (2 mg/kg), or MPZ (2 mg/kg) suspended in PBS (1.0 mL) were administered orally to colitic rats once per day. Additionally, one day after sixth treatment with each drug, the rats were killed to evaluate anti-colitic effects of the drugs. (**A**) Left panel: Photos of the distal colons of rats where serosal and luminal sides are shown separately. Right panel: Overall colonic damage was scored for each group and presented as colonic damage score (CDS). * α < 0.05, vs. DNBS control. In the inflamed distal colons (4.0 cm), (**B**) myeloperoxidase (MPO) activity was measured in addition to determining the levels of (**C**) CINC-3, (**D**) iNOS and COX-2, as well as (**E**) IL-10 using ELISA kits and Western blotting. A loading control (α-tubulin) was used for Western blot analysis of COX-2 and iNOS. The data in (**A**–**C**,**E**) are presented as mean ± SD (n = 5). Statistical significance was analyzed using a one-way ANOVA followed by Tukey’s HSD test, * *p* < 0.05, vs. DNBS control. ^#^ *p* < 0.05.

Since tCA activates GPR109A in cells, a therapeutic target whose activation is beneficial in experimental colitis [28,30,31], we examined whether tCA exerted anti-colitic effects by activating GPR109A. To do this, both tCA-GA and MPZ were co-administered to DNBS-induced colitic rats via oral gavage once per day for 6 days, and it was assessed whether the anti-colitic effects of tCA-GA were affected by preventing the activation of GPR109A using the GPR109A antagonist MPZ. The same experiment was repeated with the oral gavage of a single treatment with MPZ or tCA-GA. Consistent with the results in Figure 3, oral tCA-GA substantially alleviates colonic damage, including shortening of the colon length induced by DNBS instillation, as shown in Figure 5A (left panel showing the photos of distal colons and right panel). Change in the colon length is presented as a bar graph in Supplementary Data S3B. In parallel, DNBS-induced colonic inflammation, assessed by MPO activity and levels of inflammatory mediators (CINC-3, iNOS, and COX-2) in the distal colon, was markedly suppressed by oral tCA-GA (Figure 5B–D). Co-administration of MPZ significantly compromised the beneficial effects of tCA-GA on colonic damage and inflammation as shown in Figure 5A–D. To further examine an involvement of GPR109A activation in the anti-colitic activity of tCA, the IL-10 levels in the inflamed colon were assessed. As shown in Figure 5E, in agreement with the cellular results (Figure 4B), oral tCA-GA increased the levels of IL-10 in the inflamed colon and combined treatment with MPZ significantly suppressed the increase in the levels of IL-10 in the inflamed colon of tCA-GA-treated rats. On the other hand, a single treatment with MPZ led to an inflammatory state similar to that induced by the DNBS control (Figure 5A–E). These results indicate that tCA, generated from tCA-GA in the inflamed colon, exerted anti-colitic activity, at least partly, via GPR109A activation.

## 4. Discussion

In this study, to develop an IBD drug from natural products, tCA was selected as the lead compound based on its availability in nature and its safety and potential anti-inflammatory activity. Colon-targeted drug delivery was employed to optimize the therapeutic activity and safety of the natural product. Colon-targeted prodrugs of tCA were designed and prepared, and their colon specificity and anti-colitic activity were evaluated in vitro and in vivo. 

Conjugation of a compound with a carboxylic functional group with acidic amino acids is a feasible strategy for colon-targeted delivery of compounds [19,21]. Accordingly, tCA conjugated with glutamic acid and aspartic acid, tCA-GA, and tCA-AA acted as colon-targeted prodrugs of tCA. The conjugates lowered the distribution coefficient of tCA from 2.23 to −0.98 (tCA-GA) and −0.61 (tCA-AA), respectively. In parallel, amino acid conjugation retarded the transport of tCA via the Caco-2 cell monolayer. The conjugates liberated tCA in the cecal contents while remaining intact in the small intestinal contents. These results indicate that, following oral administration, the systemic absorption of tCA conjugated with amino acids is less effective than that of tCA during transit of the upper intestine, enabling a larger fraction of the conjugates to arrive at the large intestine, where they are converted to tCA. This was verified using an in vivo experiment, which revealed that oral tCA-GA delivered greater amounts of tCA to the cecum than oral tCA following oral gavage at an equimolar dose. 

Colon-targeted delivery of tCA potentiates its anti-colitic activity. This argument is supported by the results demonstrating that tCA-GA (equivalent to 15 mg/kg of tCA) is more effective against colonic damage and inflammation in rats than 30 mg/kg of tCA. Therapeutic potentiation might be associated with colon-targeted delivery that increases the therapeutic availability of tCA at the target site. However, a further increase in anti-colitic activity was not observed at a dose of tCA-GA equivalent to 30 mg/kg of tCA. This finding indicates that the maximal therapeutic activity of tCA-GA was reached at a low dose. Moreover, the anti-colitic activity of tCA-GA was greater than that of the current anti-IBD drug, SSZ, as depicted in Figure 3A, where tCA-GA was demonstrated to be more effective than SSZ against colonic damage and inflammation in rats. As the therapeutic use of SSZ is limited to mild to moderate IBD (especially UC), likely due to its low anti-inflammatory efficacy [4], tCA-GA may be therapeutically applicable to severe and mild to moderate IBD. 

In accordance with our hypothesis, based on the structural similarity of tCA to the GPR109A agonist, butyric acid, tCA acted as an agonist of GPR109A in the cellular assay despite its markedly lower ED_50_ than NA. This result was further verified by the data showing that the GPR109A antagonist, MPZ, effectively undermined the biological effects ascribed to tCA-mediated GPR109A activation and modulation of LPS-mediated induction of COX-2, iNOS, and IL-10 in RAW264.7 cells. Consistent with previous studies that demonstrated the beneficial effects of GPR109A agonists against experimental colitis [25,28], tCA exerted anti-colitic effects, at least partly, by activating GPR109A. This finding is supported by our data, which revealed that the anti-colitic effects of oral tCA-GA were notably compromised by the co-administration of MPZ. Accordingly, MPZ attenuated the modulatory effects of oral tCA-GA on the induction of the inflammatory mediators, COX-2, iNOS, and IL-10, which is consistent with the cellular assay results. 

Owing to the low potency of tCA for GPR109A activation, its colon-targeted delivery, which increases its therapeutic concentrations at the target site (large intestine), enables tCA to effectively activate the anti-colitic receptor. Therefore, the better anti-colitic activity of oral tCA-GA, compared to that of oral tCA, correlates well with our argument on the therapeutic mechanism of tCA against rat colitis. The data in Figure 3 show that the therapeutic concentration of tCA was markedly higher at the target site with oral tCA-GA than with oral tCA. In addition to decreasing the levels of the inflammatory mediators (COX-2 and iNOS), oral tCA-GA increased the levels of IL-10 in the inflamed colon and was more effective than oral tCA (Supplementary Data S6). 

Although immunosuppressants and glucocorticoids, applicable to severe IBD, have therapeutic limitations when administered as long-term IBD therapies (i.e., safety issues), 5-ASA-based drugs, including SSZ, have low anti-inflammatory efficacy, limiting their use in mild to moderate IBD [4]. Our data revealed that tCA-GA was more effective against rat colitis than SSZ, suggesting that the amino acid conjugate of the natural product, tCA, may be an anti-IBD drug with a broader therapeutic spectrum and improved toxicological properties. 

As tCA can be produced from the microbial metabolism of polyphenolic natural products in food in the large intestine, it is worth investigating whether tCA contributes to the anti-colitic activity of polyphenolic natural products [32] and acts as a beneficial metabolite for the health of the large intestine similarly to the GPR109A agonist, butyric acid, generated from dietary fiber in the large intestine [30,31,33]. 

## 5. Conclusions

Overall, colon-targeted delivery of tCA potentiates the anti-colitic activity of tCA and elicits anti-colitic effects superior to SSZ, a current anti-IBD drug, at least partly, by effectively activating GPR109A in the inflamed colons of rats.

## Data Availability

The data presented in this study are available in article or Supplementary Materials here.

## Supplementary Materials

The following supporting information can be downloaded at: https://www.mdpi.com/article/10.3390/pharmaceutics15010041/s1, Supplementary Data S1. Original images of western blots. Supplementary Data S2. Instrumental characterization of tCA-GA and tCA-AA. (A) FTIR spectra of tCA-GA and tCA-AA, (B) 1H-NMR spectra of tCA-GA and tCA-AA, (C) Mass spectra of tCA-GA and tCA-AA. Supplementary Data S3. Change in colon length of colitic rats treated with drugs. Three days after the induction of colitis, (A) tCA (30 mg/kg), SSZ (30mg/kg) and tCA-GA [equivalent to 15 mg/kg (L) and 30 mg/kg (H) of tCA] suspended in 1.0 mL of PBS (pH 7.4) were administered orally to rats once per day. The rats were sacrificed after the sixth dose. The length of distal colon samples was determined. (B) tCA-GA (28 mg/kg), tCA-GA (28 mg/kg) + MPZ (2 mg/kg), or MPZ (2 mg/kg) suspended in PBS (1.0 mL) were administered orally to colitic rats once per day. The rats were sacrificed after the sixth dose. The length of distal colon samples was determined. The data in A and B are represented as the mean ± SD (n = 5). * *p* < 0.05, vs. Control, ^#^ *p* < 0.05. Supplementary Data S4. Modified scoring system. Supplementary Data S5. Structures of cinnamic and butyric acids. Supplementary Data S6. IL-10 levels in the inflamed colons after the oral administration of drugs. Three days after the induction of colitis, tCA (30 mg/kg), SSZ (30mg/kg) and tCA-GA [equivalent to 15 mg/kg (L) and 30 mg/kg (H) of tCA] suspended in 1.0 mL of PBS (pH 7.4) were administered orally to rats once per day. The rats were sacrificed after the sixth dose. The tissue (distal colon) samples were subjected to ELISA and the levels of IL-10 in the tissue homogenate supernatants were determined. The data are represented as the mean ± SD (n = 5). * *p* < 0.05, vs. the DNBS control, ^#^ *p* < 0.05.
