# Supplementary material for: Colon-Targeted Trans-Cinnamic Acid Ameliorates Rat Colitis by Activating GPR109A
Source: Pharmaceutics. 2022 Dec 22;15(1):41. doi: 10.3390/pharmaceutics15010041 (PMC9865397; doi:10.3390/pharmaceutics15010041)
Supplement: Supplementary file 1 [file pharmaceutics-15-00041-s001.zip › pharmaceutics-2029028-supplementary.pdf]

Supplementary Data S1. Original images of western blots

Fig. 3 E Original Data

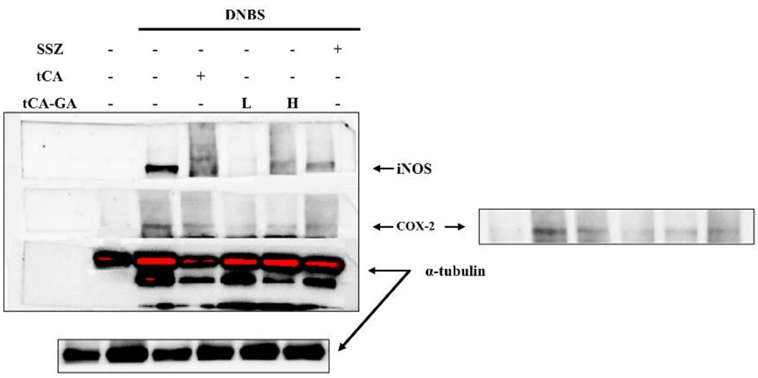

Fig. 4 C Original Data

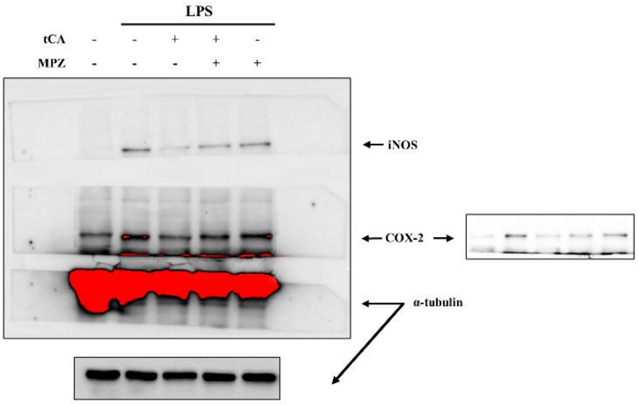

Fig. 5 D Original Data

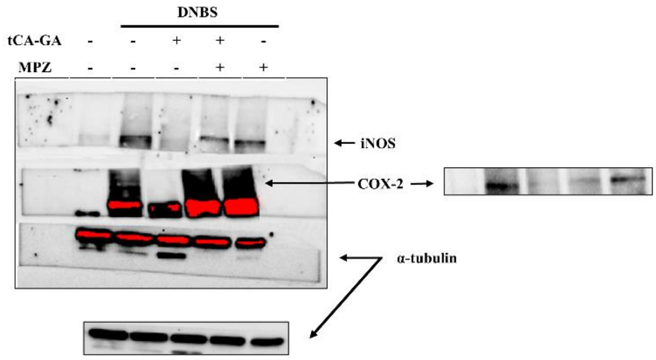

Supplementary Data S2. Instrumental characterization of tCA-GA and tCA-AA

A

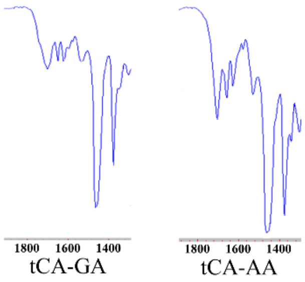

B

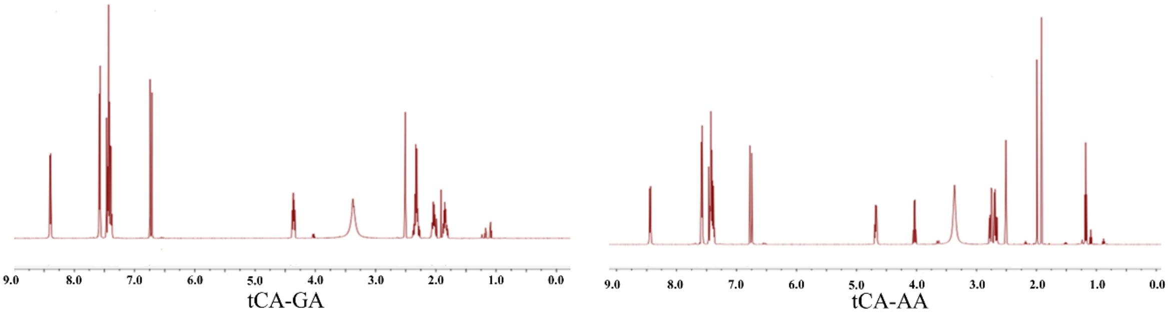

C

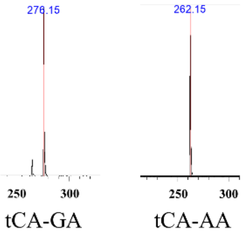

(A) FT-IR spectra of tCA-GA and tCA-AA

(B) ^{1}H-NMR spectra of tCA-GA and tCA-AA

(C) Mass spectra of tCA-GA and tCA-AA

Supplementary Data S3. Change in colon length of colitic rats treated with drugs

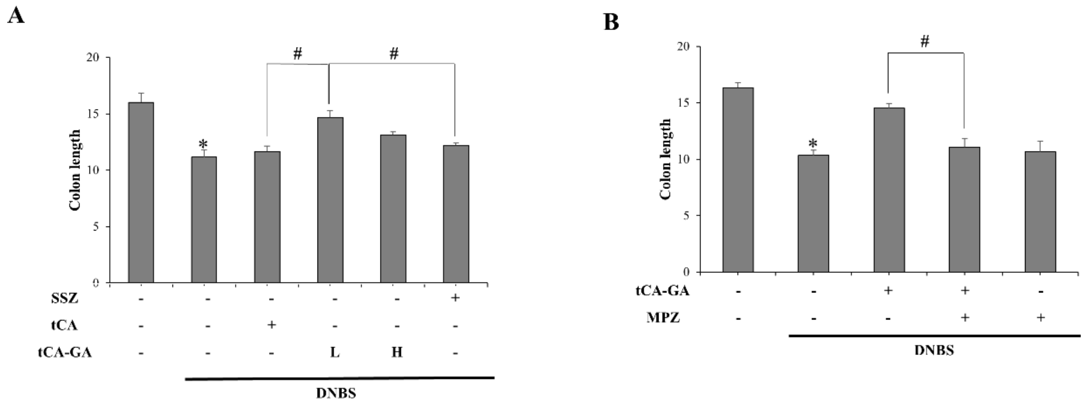

Three days after the induction of colitis, (A) tCA (30 mg/kg), SSZ (30mg/kg) and tCA-GA [equivalent to 15 mg/kg (L) and 30 mg/kg (H) of tCA] suspended in 1.0 mL of PBS (pH 7.4) were administered orally to rats once per day. The rats were sacrificed after the sixth dose. The length of distal colon samples was determined. (B) tCA-GA (28 mg/kg), tCA-GA (28 mg/kg) + MPZ (2 mg/kg), or MPZ (2 mg/kg) suspended in PBS (1.0 mL) were administered orally to colitic rats once per day. The rats were sacrificed after the sixth dose. The length of distal colon samples was determined. The data in A and B are represented as the mean ± SD (n = 5). $^{*}P < 0.05$, *vs.* Control, $^{\#}P < 0.05$.

Supplementary Data S4. Modified scoring system

Score	Feature
0	normal appearance
1	localized hyperemia but no ulcer
2	linear ulcers without significant inflammation
3	2–4 cm site of inflammation and ulceration
4	serosal adhesion to other organs, 2–4 cm site of inflammation and ulceration
5	stricture, serosal adhesion involving several bowel loops, <4 cm site of inflammation and ulceration

Supplementary Data S5. Structures of cinnamic acid and butyric acid

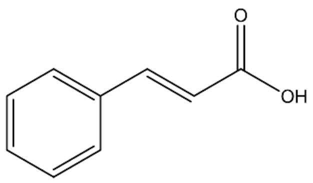

Cinnamic acid

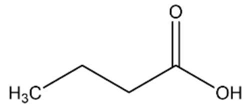

Butyric acid

Supplementary Data S6. Levels of IL-10 in the inflamed colon after oral administration of drugs

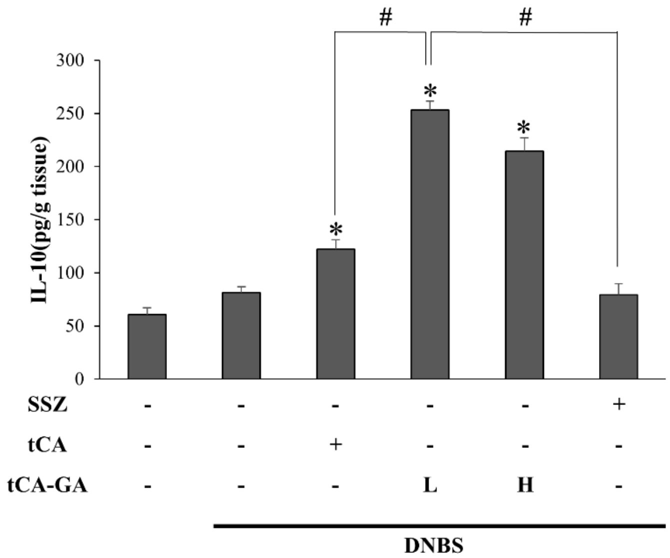

Three days after the induction of colitis, tCA (30 mg/kg), SSZ (30mg/kg) and tCA-GA [equivalent to 15 mg/kg (L) and 30 mg/kg (H) of tCA] suspended in 1.0 mL of PBS (pH 7.4) were administered orally to rats once per day. The rats were sacrificed after the sixth dose. The tissue (distal colon) samples were subjected to ELISA and the levels of IL-10 in the tissue homogenate supernatants were determined. The data are represented as the mean ± SD (n = 5). $^{*}P < 0.05$, *vs.* the DNBS control, $^{\#}P < 0.05$.
